# Sinonasal Headaches and Post-Operative Outcomes after Septoplasty in Patients with Nasal Septal Deviation

**Published:** 2011

**Authors:** Ali Ghazipour, Hassan Abshirini, Mahmood Hekmat shoar, Sara Pursalehan

**Affiliations:** 1 Department of otorhinolaryngology, Ahwaz Jundishapur University of Medical Sciences, Ahwaz, Iran; 2 Department of otorhinolaryngology, Ahwaz Jundishapur University of Medical Sciences, Ahwaz, Iran; 3 Department of otorhinolaryngology, Ahwaz Jundishapur University of Medical Sciences, Ahwaz, Iran; 4 General Physician

**Keywords:** Follow-up, Headache, Septoplasty, Septal Deviation, Sinonasal

## Abstract

**Introduction:**

Investigators believe that anatomical abnormalities in the sinonasal region can be the cause of some chronic and refractory headaches that may respond well to surgical intervention. This study presents the prevalence of headache in patients with nasal septal deviation and their response to surgical treatment over a 2-year follow-up period.

**Materials and Methods:**

This descriptive and prospective study was conducted on 98 patients with nasal septal deviation who underwent septoplasty surgery in the Imam Hospital in Ahwaz. Preoperative information was acquired by asking the patients and by completing SNOT-20 questionnaires by patients. After the surgery, information about changes in the quality of headache in patients with dominant contact points in preoperative nasal endoscopy whose headache responded to topical anaesthesia with lidocaine 2%+naphazoline 0.5% was collected over a 2-year follow-up. Final data were analyzed by SPSS and descriptive statistics.

**Results:**

Ninety-eight patients were studied, comprising 58.2% men and 41.8% women. They ranged in age between 18 and 46 years (mean=24). Nasal obstruction (72.4%), snoring (58.1%), headache (46%) and epistaxis (17.3%) were the most frequent preoperative symptoms. The most common site of the headache was the frontal region (68.8%). Patients' headache was bilateral in 71.1% of cases. In 82.2% of patients, headache lasted less than four hours a day. The headache was pulsatile in 53.3%, sharp in 31.2% and compressive in 15.5% of cases. In the post-operative assessment, despite gradual decline in the referral patients for follow-up, a notable and gradual recovery in patients’ headache was seen with 82.8% of the patients reporting complete or partial recovery of the headache at the end of the 2-year follow-up.

**Conclusion:**

Headache is one of the most common symptoms in patients with nasal anatomical abnormalities such as septal deviation and usually responds well to surgical treatment. More studies with long-term follow-ups seems to be inevitable to determine the relationship between headaches and nasal anatomical abnormalities, accurate surgical results in patients’ recovery and the recurrence rate of headaches.

## Introduction

The nasal septum is straight at birth and will remain in this state throughout infancy and the early years of childhood. At times, septal deviation is congenital, usually caused by traumas suffered at the time of delivery (1). The nasal septum seems to lose its central position as the person grows, and this trend will assume a progressive nature with growth (2).

The nasal septum is the first contact point of the airways and the inhaled air, the deviation of which causes air turbulence. Marked nasal septal deviation causes disturbances in the airways, resulting in dryness, crusting and dysplastic changes in parts of the mucus where airflow has increased. It can also cause disturbances in the cycle of normal nasal secretions. Nasal septal deviation can also cause such symptoms as nasal congestion, epistaxis, snoring, headaches and oral breathing (3).

When septal deviation is parallel with the infundibular part of the lateral wall, it will cause changes to ventilation and it will also cause contact points between opposite mucosal surfaces. Contact points may disable ciliary activity and prevent mucal drainage from paranasal sinuses, and may hence cause an accumulation of secretions in sinuses. Thus, any anatomical abnormalities or pathologies of the nose can manifest itself as a clear condition such as chronic sinusitis, halitosis or headaches (4, 5).

Sinonasal headaches are attributed to the presence of contact points between opposite mucosal surfaces in the sinonasal region, such as contacts between the nasal septum and the superior or middle conchae or with inner walls of the ethmoid sinus. These contact points can intensify primary headaches or cause secondary headaches, which have recently been added to the International Classification of Headache Disorders. 

As defined, these headaches are characterized by frequent localized headaches, particularly in the periorbital region, the inner wall of the eye, and the temporal region, and also in the forehead and the upper jaw, and are accompanied by the presence of contact points in the sinonasal region (6). 

The causative mechanism is triggered when sinonasal receptors, upon stimulation, transfer the pain using Substance P to the sensory nucleus of the fifth nerve, which receives the sensory fibers of the face and the neck. These fibers eventually reach the same cortical region; thus, pain signals upon reaching the sensory cortex can be falsely localized and can cause refractory pains in other parts of the head and face (4,5). These contact points have also been recognized in recent researches to trigger migraine headaches, so surgical treatment of these points can lead to improvements in such headaches (7,8).

Headaches are a common compliant of those who visit ENT specialists, the cause of which can remain controversial or even unidentified. Since researches have shown that anatomical abnormalities of the nose, like septal deviation, can cause chronic and refractory headaches, determining the prevalence of headaches in such patients and a follow-up of their response to surgical treatments can be a good reason for higher attention to and better examination of the patients, especially those who have been examined repeatedly without having identified any specific cause for their headaches. Further, in view of the role of surgery in improving the anatomical abnormalities of the nose and the ensuing symptoms, ENT examinations should be added to the list of required examinations for patients with headaches (4,5). 

Therefore, in this study, we tried to investigate the prevalence of headaches, and also their quality, intensity and the duration in patients suffering from nasal septal deviation, and make an assessment of patients’ improvement after surgery.

## Materials and Methods

In this descriptive, prospective study, 98 patients diagnosed with nasal septal deviation and hospitalized for septoplasty surgery at the Imam Hospital, Ahwaz, Iran, in 2008, were studied. All these patients had suffered from nasal septal deviation and the accompanying symptoms for over five years and had all undergone symptomatic drug treatment. The diagnosis of nasal septal deviation had been proposed by examination and anterior rhinoscopy using nasal speculum, and was finalized by the CT-Scan of paranasal sinuses. All those for whom there were findings for acute or chronic rhinosinusitis based on clinical examinations or PNS CT-scan were excluded from this study. After the selection of the patients, demographic data, such as age and sex, were collected and recorded. All the participants completed the SNOT-20 forms prior to the surgery. 

Those suffering from headache completed the form again at 3-moth, 6-month, 1-year and 2-year intervals after the surgery. (It should be noted that SNOT-20 is an international, standard questionnaire with 20 different items concerning sinonasal symptoms and their effect on the patient’s quality of personal and social life. 

Each item is scored on a scale of 0 to 5, with the lowest for “no problem” case, and the highest for “problem as bad as it can be” case,which are averaged to give a total score.Lower scores indicate a better perception of quality of life). 

Those who suffered from headaches underwent endoscopy, and then local anesthesia of the inside of the nose with lidocaine +2% and naphazoline 0.1%. Those patients who had clear contact points in their nasal septa in the endoscopy and whose headaches had responded to local anesthesia were followed up postoperatively. 

Then all the patients underwent septoplasty under general anesthesia using the submucosal resection technique. After surgery, all patients had interior nasal packs, which were removed 3-5 days later. 

All the data obtained from SNOT-20 forms completed before and after the surgery were statistically analyzed by SPSS (v. 17). 

## Results

***Result***s 

In this study, out of the 98 participants, 57 were men (58.2%) and 41 were women (41.8%). They ranged between 18 and 46 years in age (mean: 24 years). Nasal congestion was the most common compliant at 72.4% and headaches were the third most common at 46% (Table 1).

**Table 1:** The prevalence of preoperative complaints in the 98 patients in this study

**Prevalence**	**Absolute** **(number)**	**Relative** **(percentage)**
**Symptoms**		
**Nasal congestion**	71	72.4
**Oral breathing**	62	63.2
**Headaches**	45	46
**Epistaxis**	17	17.3

Out of the 45 patients who had distinct contact points in endoscopy and whose headache had responded to local anesthesia, 31 patients (68.8%) complained of frontal headaches, and only three patients (6.6%) reported occiput or vertex pains (Table 2).

Headaches were bilateral in 32 patients (71.1%) and unilateral in 13 (28.9%). As for the duration, 37 patients (82.2%) reported the headaches to last less than for hours per day. 

Six people (12.4%) reported this as between four and eight hours per day, and two people (4.4%) as more than eight hours per day (with an average of 12 hours). The pains were pulsatile in 24 patients (53,3%), sharp in 14 (31.2%) and compressive in seven (5.5%).

In the follow-up after three months after the surgery, all the 45 patients participated, out of whom 18 patients (40%) reported full treatment of the headaches.

**Table 2:** The prevalence of preoperative headache sites in 45 patients

**Prevalence**	**Number of patients**
**Pain site**	
**Frontal region**	31
**Periorbital**	20
**Temporal region**	17
**Face**	14
**Parietal region**	5
**Vertex**	3
**Occiput**	3

Fifteen patients (33.3%) were satisfied of decreased intensity and/or frequency of headaches, and, ultimately, 11 patients (24.5%) did not report any reduction in the intensity, frequency or quality of their headaches. 

Only one patient complained of intensified headaches after the surgery. In the long-term follow-ups, despite the gradual decline in the number of participants, there was a gradual rise in the improvement of the headaches, so that out of the 35 participants in the last follow-up, after two years, 29 patients reported complete recovery or pain reduction, accounting for 82.8% (Table 3).

**Table 3:** The rate of changes in quality of headache in postoperative follow-up

**Follow-up time**	**3 moths** ** post-op**	**6 moths ** **post-op**	**1 year ** **post-op**	**2 years ** **post-op**
**Change in headache quality**	**number of patents**	**number of patents**	**number of patents**	**number of patents**
**Full recovery**	18	17	16	16
**Reduced headache**	15	15	16	13
**No change**	11	10	8	6
**Headache intensification**	1	1	0	0
**All follow-up participants**	45	43	40	35

## Discussion

Sinonasal headaches can prove quite difficult to study and examine because of special nature of condition, accompanying illnesses and problems occurring during follow-up period.

Out of the 98 patients in our study, 57 were men (58.2%) and 41 were women (41.8%) with mean age 24 years. According to this study, 71 people (72.4%) suffered from nasal congestion, 62 (63.2%) from oral breathing, 45 (46%) from headaches, and 17 (17.3%) from epistaxis.

As far as the prevalence of symptoms is concerned, these finding correspond to those found by Low and Willatt in their pre-septoplasty study on 75 people. According to their study, 57.3% of the patients suffered from oral breathing, 48% from headaches, and 21.3% from epistaxis (9). Out of the 45 patients in our study who complained of headaches, 31 (68.8%) people reported the frontal region, 20 (44.4%) the periorbital region, 17 (37.7%) the temporal region and 14 (31.1%) the face as the site of the headaches. Headaches in all the sites mentioned were bilateral in 71.1% of patients. In a study conducted by Harly and Powitzky in the US, 71 patients who complained of headaches and whose nasal anatomical abnormalities, such as nasal septal deviation, had been confirmed were studied. Their headaches were reported to be in the frontal region in 73%, in the periorbital region in 38%, in the temporal region in 28% and in the face in 26% of cases. Also, 78% of the patients had bilateral headaches according to the case histories (11). Overall, these finding correspond to ours. Based on these statistics, most patients experience their headaches in more than one anatomical location. As for the quality of the pains, 24 people (53.3%) reported pulsatile pains, 14 (31.2%) sharp pains, and seven (15.5%) compressive pains. In the study conducted by Behin and Bigal in the US, headaches caused by contact points in the sinonasal region have been reported to be pulsatile, sharp or compressive (7).

In our study, three months after the surgery, 18 patients (40%) reported full recovery and 15 (33.3%) reported a decline in pain intensity; these numbers rose in the last follow-up, two years after the surgery, with 35 participants, to 16 (45.7%) for full recovery and 13 (37.1%) for reduced intensity. In the Low and Willatt’s study in the UK, in the 2-year follow-up, recovery reached the rate of 33.3% (9). In the Behin and Bigal's study in 2004, after the surgery 91% of patients reported reduced headache intensity and 85% reduced headache frequency (8). In another study conducted by the same researchers in 2006, in a 10-year follow-up after the surgery, 30% of the patients had full recovery, and 35% had significant recovery (7). 

In another study by Ramadan et al, in a 3-month follow-up, 60% of people who had undergone surgery were cured of headache (12). 

In a study by Welge-Luessen et al in Basel, Switzerland, in a 10-year follow-up, 65% of the patients reported complete disappearance of the headaches or marked improvements in the symptoms. Interestingly, two patients in the study who wre asymptomatic 7-8 years after the surgery experience a relapse of headaches (13). In other studies, too, the removal of the sinonasal factors, such as nasal septal deviation, had led to complete disappearance of headaches or marked improvement in most patients (10,14). As can be seen in the above studies, in the follow-ups after the surgery, patients had different responses to the surgery in terms of reduction in symptoms, which may have been caused different natures of the problems and the accompanying conditions. Also, as said before, contact points can cause secondary headache disorders or intensify primary headaches, and, therefore, removing the contact points can lead to relative recovery, but because the primary cause of the condition persists, the symptoms may continue to exist.

## Conclusion

In view of the numerous studies on patients with nasal anatomical abnormalities, such as nasal septal deviation, having undergone septoplasty show that headaches are one of the most common preoperative complaints, and also in view of the fact that these headaches respond well to surgical treatments, postoperative follow-ups can identify both the relationship between headache symptoms and nasal anatomical abnormalities and the effect of surgery on the recovery rate of headache in patients. On the other hand, in view of the relapse of headaches in a few cases in some recent long-term studies, 10-20-year, long-term follow-ups are recommended to identify the exact effect of the surgery on these patients and the rate of relapse.
